# Supplementary material for: MmcA is an electron conduit that facilitates both intracellular and extracellular electron transport in Methanosarcina acetivorans
Source: Nat Commun. 2024 Apr 17;15:3300. doi: 10.1038/s41467-024-47564-2 (PMC11024163; doi:10.1038/s41467-024-47564-2)
Supplement: Supplementary file 1 — Supplementary Information [file 41467_2024_47564_MOESM1_ESM.pdf]

Supplementary Information

Title: MmcA is an electron conduit that facilitates both intracellular and extracellular electron transport in *Methanosarcina acetivorans*

Authors: Dinesh Gupta[1], Keying Chen[2], Sean J. Elliott[2], Dipti D. Nayak[1*]

Department of Molecular and Cell Biology, University of California, Berkeley, CA, USA[1]; Department of Chemistry, Boston University, Boston, MA, USA[2]

*Address correspondence to:

Dipti D. Nayak (dnayak@berkeley.edu)

Department of Molecular and Cell Biology, 1 Barker Hall #3204, University of California, Berkeley, CA 94720-3204

Tel: 510-664-5267

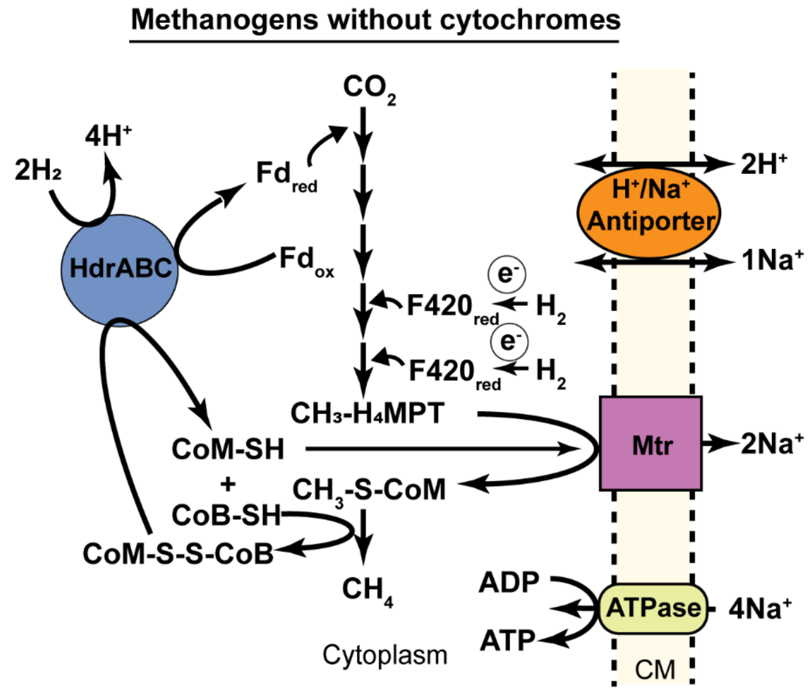

Supplementary Figure 1: Mtr (N^5-methyl H_4MPT: CoM Methyltransferase) dependent energy conservation in methanogens without cytochromes. Methanogens without cytochromes, typically perform hydrogenotrophic methanogenesis, where in CO_2 is reduced to CH_4 using electrons derived from H_2 gas. An electron bifurcating HdrABC complex in the cytosol uses H_2 gas to reduce ferredoxin for the first step of methanogenesis and regenerate coenzyme M (CoM-SH) and coenzyme B (CoB-SH) from the CoM-S-S-CoB heterodisulfide produced during the last step of methanogenesis. Reduced F420 is generated by F420-dependent hydrogenases in the cytosol. The methyl transfer reaction catalyzed by Mtr (N^5-methyl H_4MPT: CoM Methyltransferase) occurs at the membrane and is coupled to the translocation of Na^+ ions required for energy conservation. Reduced Ferredoxin (Fd_{red}), reduced coenzyme F420 ($F420_{red}$), cytoplasmic membrane (CM, shown by dotted lines), sodium gradient (Na^+), proton gradient (H^+), heterodisulfide reductase complex (Hdr), tetrahydromethanopterin (H_4MPT). Figure is adapted from (1).

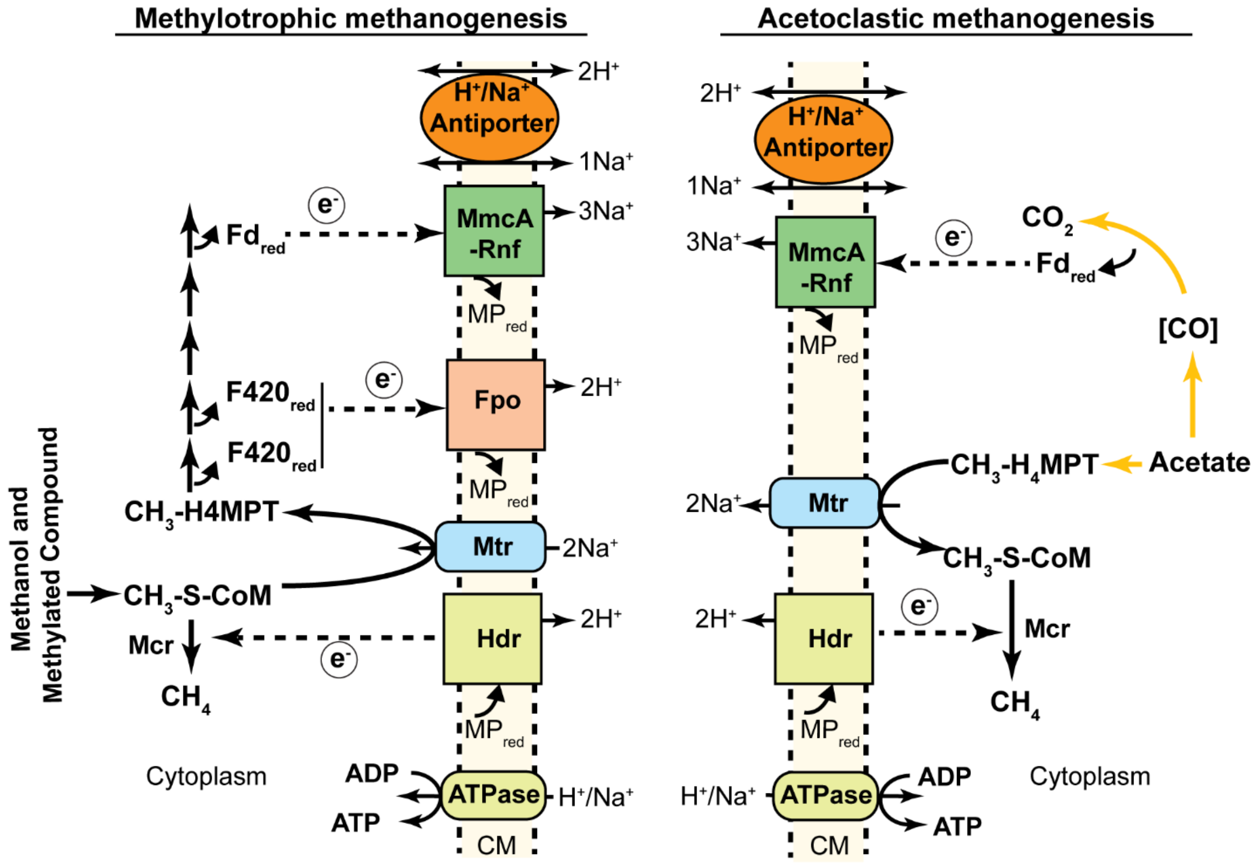

Supplementary Figure 2: Role of MmcA-Rnf complex in methane metabolism. During growth on methylated compounds or methylotrophic methanogenesis (black arrows on left) and on acetate or acetoclastic methanogenesis (yellow arrows on right) in cytochrome-containing methanogens like *Methanosarcina acetivorans*, reduced ferredoxin is generated. In all these scenarios, electrons from reduced ferredoxin enter the electron transport chain through the MmcA-containing **R**hodobacter **n**itrogen **f**ixation complex (MmcA-Rnf). Reduced Ferredoxin (Fd_{red}), reduced coenzyme F420 ($F420_{red}$), cytoplasmic membrane (CM), sodium gradient (Na^+), proton gradient (H^+), F420 dehydrogenase complex (Fpo), heterodisulfide reductase complex (Hdr), N^5-methyl H_4MPT: CoM Methyltransferase (Mtr), Methyl-coM reductase (Mcr), tetrahydromethanopterin (H_4MPT) and coenzyme M (CoM-SH). The dotted-line arrows show electron donors to specific membrane complexes of ETC or to the specific step of methanogenesis. In the absence of the MmcA-Rnf complex, reduced ferredoxin can be oxidized by alternate pathways such as the HdrABC complex as shown before (2, 3).

1

MVIMNRLNLL VSGVAVLLLL AAGA**Y**SSLGY SGNDAIASHY MTKGEWSDSV

51 (1)

CGGCHFGVYE NVNNSYHVQV NMSRWSPLTN FDLETSGEEE WVKKFGMYHP

101 (2)

GGGPLAKYGI DIDCMMCHEK YGLYDFDARA EAIANGDFAN ANSLAVANFS

151 (3)

ATAQSDPLHL FVYTANVLTP YPLLIVFHDA VNGAPISCAQ RCHRIDVETS

201 (4)

AVMWADEEDF EESDAHAANG VECTECHHTE AFIITSDHQI GRGNTSGTPD

251 (5) (6)

LPDSHYDDTM RSCDDAECHA GISHGPFADS HMEFLACEAC HTPELPGGDL

301

PGGNVLESFS WQNGEREDVY RDSDFQPALA WYNGNFGDVL PSVDTRNDTD

351

VKVTPFNNIT GTWWDAGTDP EVLANPNTSI STGDPIPVQY VKAADANGDG

401

EVTVEEMQAY DADGDGEADY PNAVLRTVEL YYQVAHSIVS SDIGLADPYT

451 (7)

CKDCHGNEAV IDWAALGYEQ DPGGESSAVK SIAVTYDKPRP VEVETEPAL

Supplementary Figure 3: Amino acid (aa) sequence of the MmcA open reading frame (ORF) from *Methanosarcina acetivorans*. The 500 aa long apo-MmcA (encoded by *MA0658*) contains, a N-terminal Sec signal peptide (1-24 aa; shown in purple), heme binding motifs (underlined in red). MmcA contains five canonical heme binding motifs (CXXCH; 1, 2, 4, 6 and 7) and two heme binding motifs with extended gap between cysteine residues (3 and 5). Holo-MmcA is predicted to be 476 aa long protein (the predicted N-terminal amino acid in Bold) with seven covalently attached hemes.

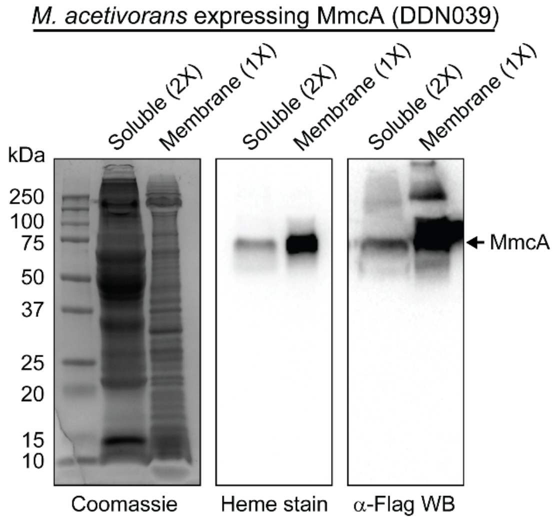

Supplementary Figure 4: MmcA is primarily a membrane-associated protein. Coomassie, heme staining and Western blot (WB) with anti (α)-Flag antibody of the soluble and membrane fractions of *M. acetivorans* with the C-terminal 3×FLAG tagged MmcA-overexpression vector (DDN039). Twice the amount of the soluble fraction (2X, ~40 µg) compared to the membrane fraction (1X, ~20 µg) was loaded to visualize the small amount of MmcA in the soluble fraction. Based on the intensity of the heme stain and the Western blot, we can conclude that MmcA is primarily present in the membrane-fraction of cells. Data shown in figure is from one biological replicate. Source data are provided as a source data file.

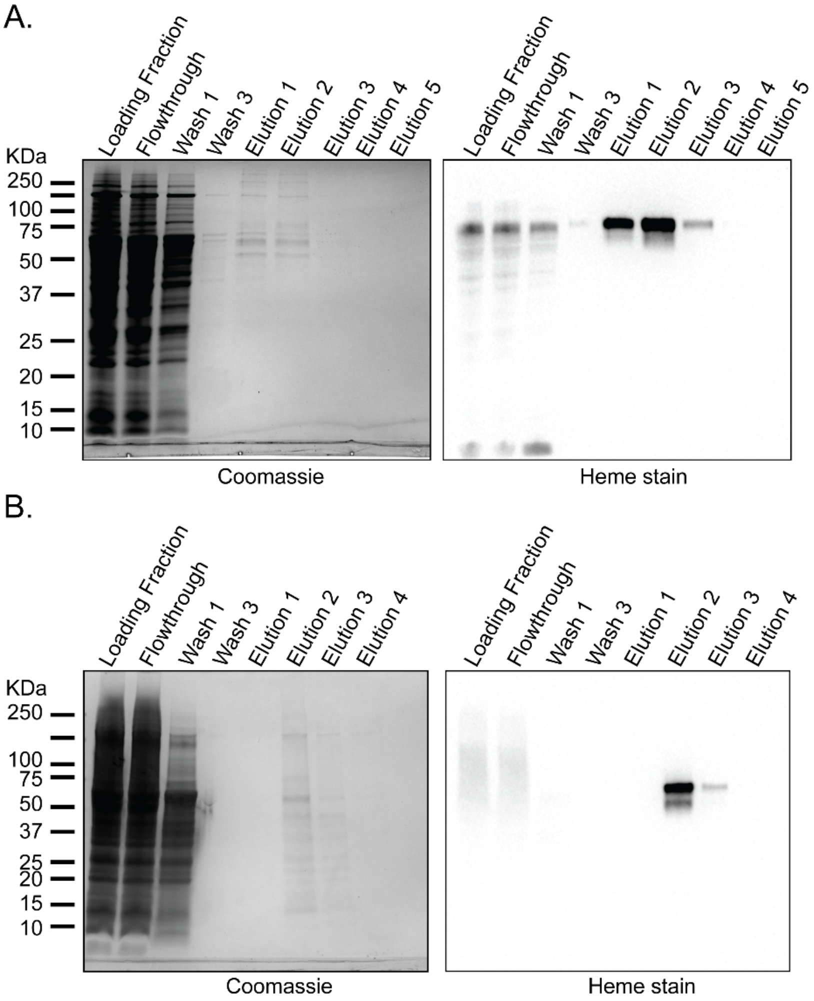

Supplementary Figure 5: Affinity purification of C-terminal twin-Strep and 3×FLAG tagged MmcA using streptactin resin. Coomassie and heme staining of different fractions during MmcA purification using streptactin resin with **(A)** phosphate buffer (pH 8) and **(B)** Tris-buffer (pH 7.4). Trace amounts of MmcA was detected by heme staining. Data shown in figure 5A, and B are from one biological replicate. Source data are provided as a source data file.

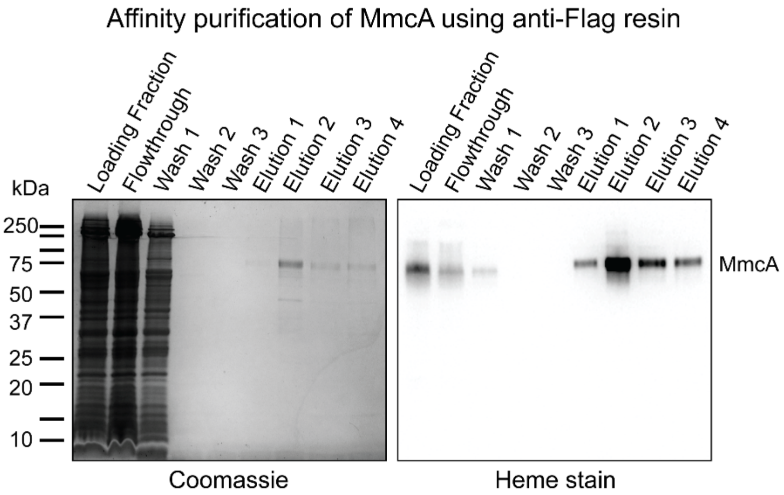

Supplementary Figure 6: Affinity purification of C-terminal twin-Strep and 3×FLAG tagged MmcA using anti-Flag resin. Coomassie and heme staining of different fractions during MmcA purification using anti-Flag resin and Tris-buffer (50 mM Tris-HCl, 150 mM sodium chloride, pH = 7.4). A distinct MmcA band was visible in both Coomassie and heme staining. Data shown in figure is from one biological replicate. Source data are provided as a source data file.

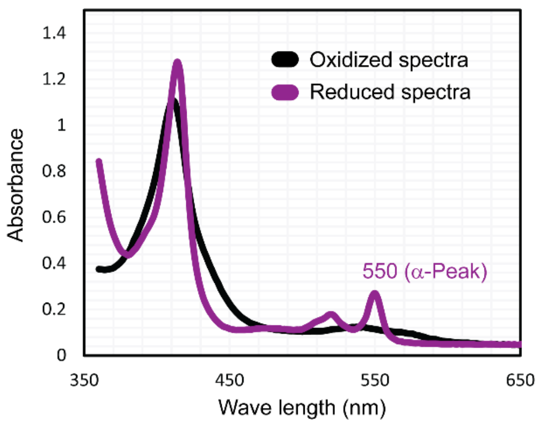

Supplementary Figure 7: Pyridine hemochrome assay shows 550 nm α-Peak for MmcA. Oxidized (black) and reduced (purple) spectra of MmcA in pyridine hemochrome assay. Data shown are representative of two experiments. Source data are provided as a source data file.

1
MVIMNRLNLL VSGVAVLLLL AAGA**Y**SSLGY SGNDAIASHY MTKGEWSDSV
51
CGGCHFGVYE NVNNSYHVQV NMSRWSPLTN FDLETSGEEE WVKKFGMYHP
101
GGGPLAKYGI DID**CMMCH**EK YGLYDFDARA EAIANGDFAN ANSLAVANFS
151
ATAQSDPLHL FVYTANVLTP YPLLIVFHDA VNGAPIS**CAQ** **RCH**RIDVETS
201
AVMWADEEDF EESDAHAANG VE**CTECH**HTE AFIITSDHQI GRGNTSGTPD
251
LPDSHYDDTM RS**CDDAECH**A GISHGPFADS HMEFLA**CEAC** **H**TPELPGGDL
301
PGGNVLESFS WQNGEREDVY RDSDFQPALA WYNGNFGDVL PSVDTRNDTD
351
VKVTPFNNIT GTWWDAGTDP EVLANPNTSI STGDPIPVQY VKAADANGDG
401
EVTVEEMQAY DADGDGEADY PNAVLRTVEL YYQVAHSIVS SDIGLADPYT
451
CKDCHGNEAV IDWAALGYEQ DPGGESSAVK SIAVTYDKPR PVEVETEPAL
501
DDDDKGGGSG GGSGGDYKDH DGDYKDHDID YKDDDDKGSA ASWSHPQFEK
551
GGGSGGGSGG GSWSHPQFEK SG

Supplementary Figure 8: Mass spectrometry analyses of MmcA. Amino acid (aa) sequence of MmcA (encoded by *MA0658*) with tandem affinity purification (TAP) tag (underlined), a predicted N-terminal Sec signal peptide (1-24 aa; shown in purple), heme binding motifs (bold red) and the predicted N-terminal amino acid after Sec signal processing (bold black) are shown. Peptides detected by LC-MS/MS analyses of MmcA (two independently purified samples) digested with either trypsin or chymotrypsin are highlighted in cyan and yellow respectively. Peptides detected under both conditions are highlighted in green.

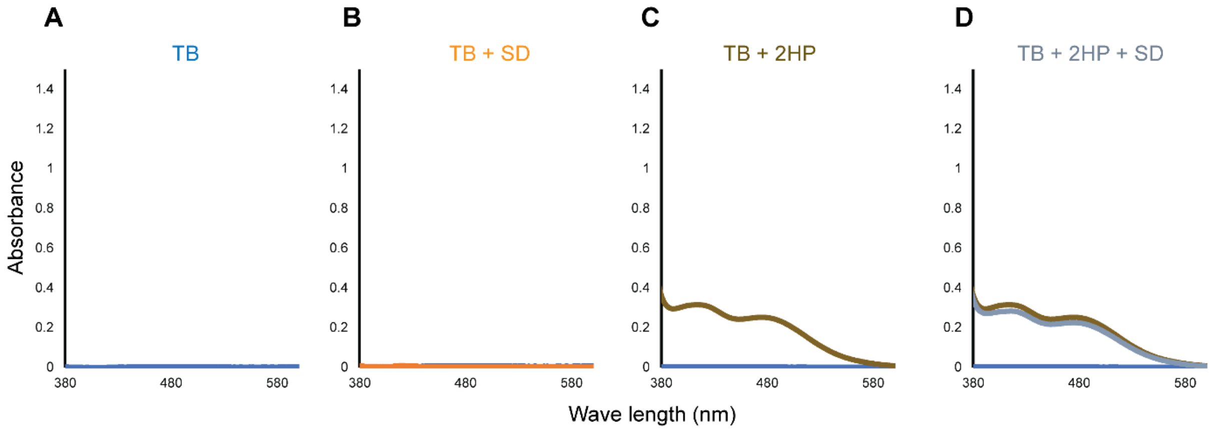

Supplementary Figure 9: Protein-free controls for MmcA assays with 2-hydroxyphenazine. (**A)** Spectra observed for Tris-buffer (TB), **(B)** TB plus sodium dithionite (SD), (**C**) TB plus 2-hydroxyphenazine (2HP), and **(D)** TB plus 2HP and SD. Data shown are representative of two replicates. Source data are provided as a source data file.

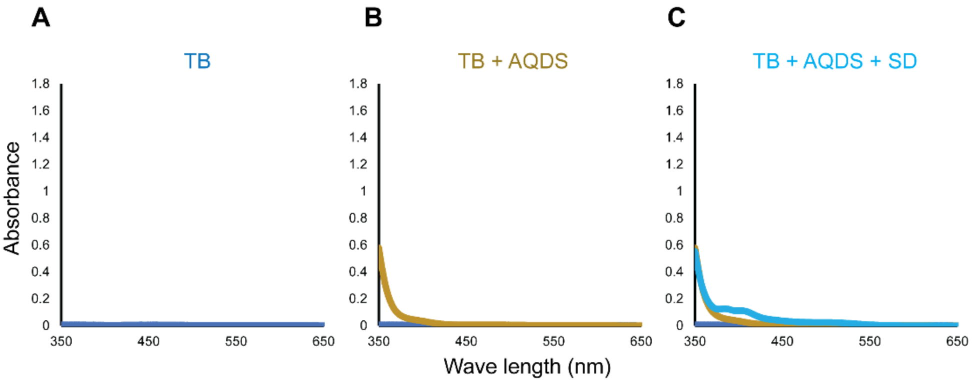

Supplementary Figure 10: Protein-free controls for MmcA assays with anthraquinone-2, 6 disulfonate (AQDS). (A) Spectra observed for Tris-buffer (TB), **(B)** TB plus AQDS, (**C**) TB plus AQDS and sodium dithionite (SD). Data shown are representative of two replicates. Source data are provided as a source data file.

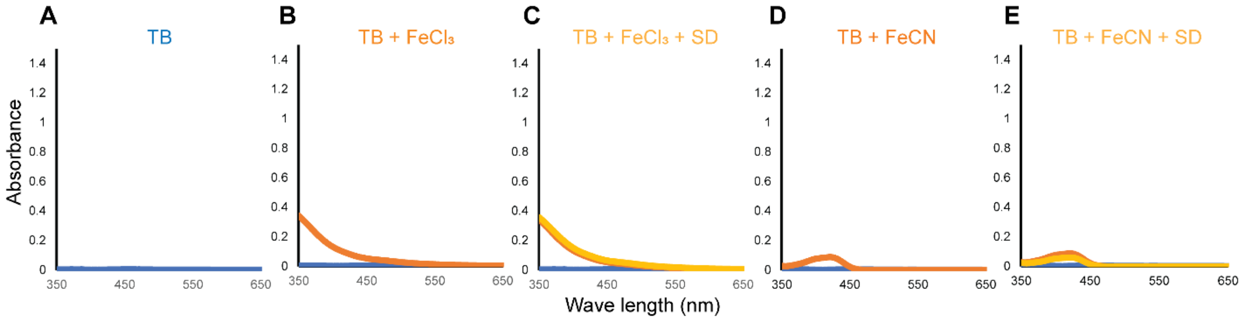

Supplementary Figure 11: Protein-free controls for MmcA assays with ferric chloride and ferricyanide in UV-visible spectral analyses. (A) Spectra observed for Tris-buffer (TB), (**B**) TB plus ferric chloride ($FeCl_3$), (**C**) TB plus $FeCl_3$ and sodium dithionite (SD), (**D**) TB plus ferricyanide (FeCN), and (**E**) TB plus FeCN and SD. Data shown are representative of two replicates. Source data are provided as a source data file.

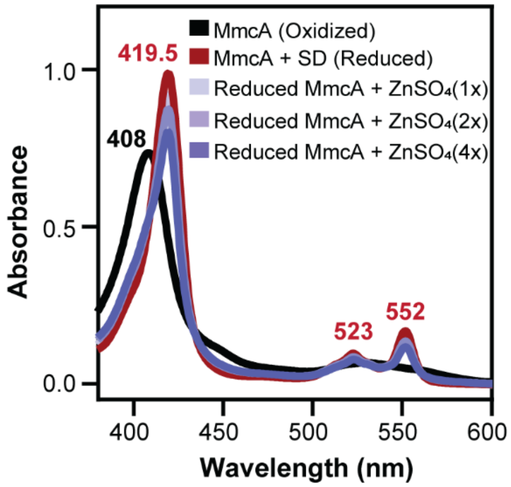

Supplementary Figure 12: MmcA cannot donate electrons to zinc (II) sulfate. Spectral analysis of MmcA mediated reduction of zinc (II) sulfate ($ZnSO_4$). Addition of $ZnSO_4$ [100 μM (1x), 200μM (2x), 400 μM (4x)] to MmcA reduced with sodium dithionite (SD; red) could not re-oxidize MmcA as the characteristic Soret peak of reduced-MmcA (red) did not shift back to the Soret peak at 408 nm indicative of the oxidized MmcA (black). Data shown are representative of two experiments. Source data are provided as a source data file.

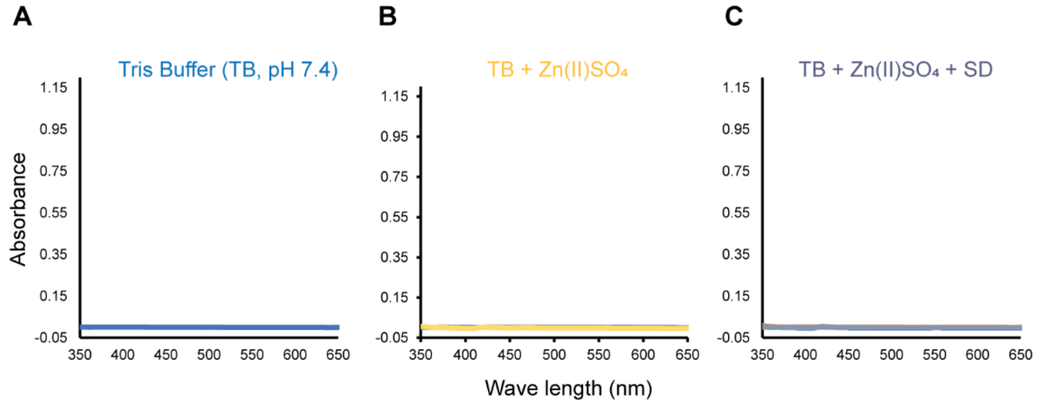

Supplementary Figure 13: Protein-free controls for MmcA assays with zinc (II) sulfate in UV-visible spectral analyses. (**A**) Spectra observed for Tris-buffer (TB), (**B**) TB plus zinc (II) sulfate ($ZnSO_4$), (**C**) TB plus $ZnSO_4$ and sodium dithionite (SD). Data shown are representative of two replicates. Source data are provided as a source data file.

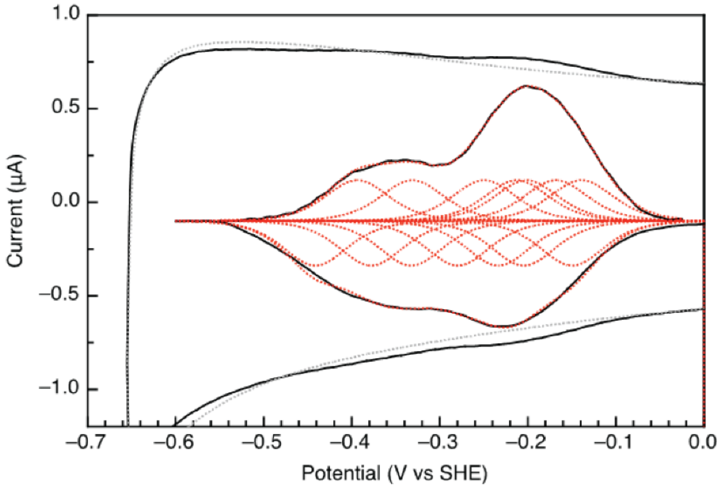

Supplementary Figure 14: Electrochemical analysis of MmcA. Non-turnover MmcA voltammogram (black solid). Gray dotted line shows the ITO electrode baseline. The background-subtracted non-turnover MmcA voltammogram (black solid) with fitting (red dotted) are shown in the middle of the graph. Cyclic voltammetry (CV) recorded at pH 7.4, 10°C, and a scan rate of 20 mV/s.

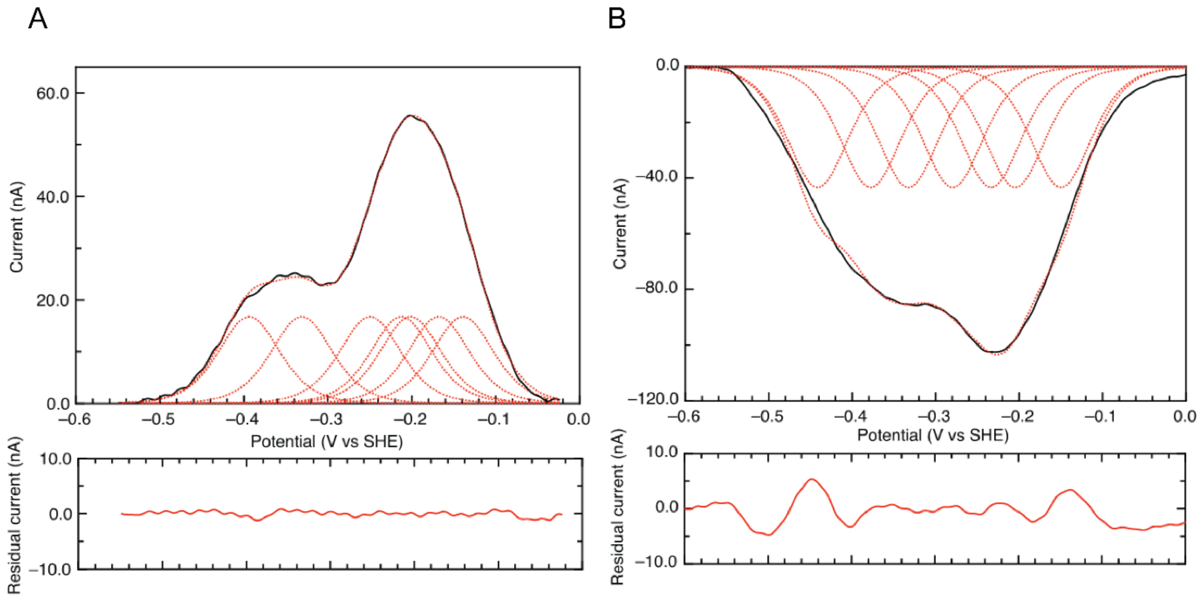

Supplementary Figure 15: Electrochemical analysis of MmcA. Residuals for fits of the **(A)** oxidative and **(B)** reductive scans of MmcA. Cyclic voltammetry (CV) was recorded at pH 7.4, 10°C, and 20 mV/s.

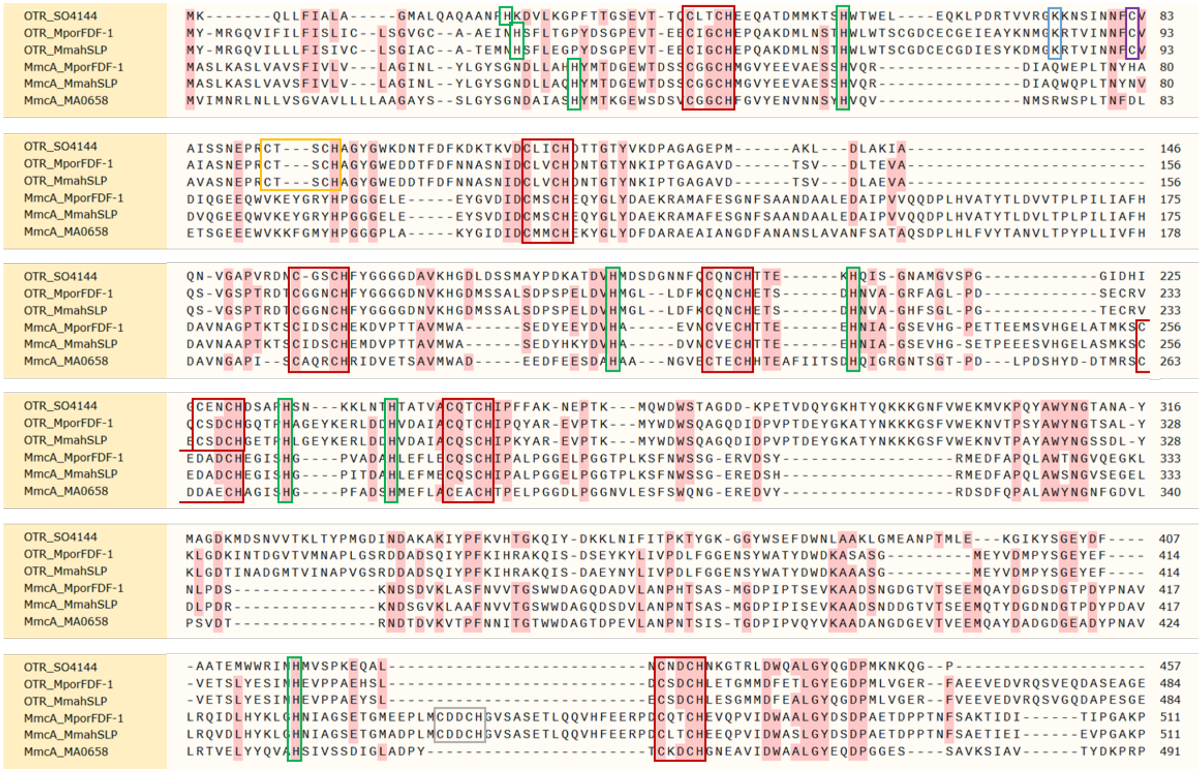

Supplementary Figure 16: Protein alignment of MmcA and octaheme tetrathionate reductase (OTR) homologs. Alignment of octaheme tetrathionate reductase (OTR) from *Shewanella oneidensis* MR-1 (OTR_SO4144, (4)), MmcA from *M. acetivorans* (MmcA_MA0658), OTR and MmcA from *Methanohalophilus mahii* strain SLP (MmahSLP) as well as OTR and MmcA from *Methanohalophilus portucalensis* strain FDF-1 (MporFDF-1). The conserved features of the OTR catalytic site i.e., the second heme-binding motif (orange box), the lysine ligand (blue box) and the cysteine residue (purple box) are shown. Seven bis-His ligated heme-binding motifs (red box) and their distal histidine ligands (green box) in OTR and MmcA are also shown. An additional heme binding motif in MmcA from MmahSLP and MporFDF-1 (gray box) is highlighted.

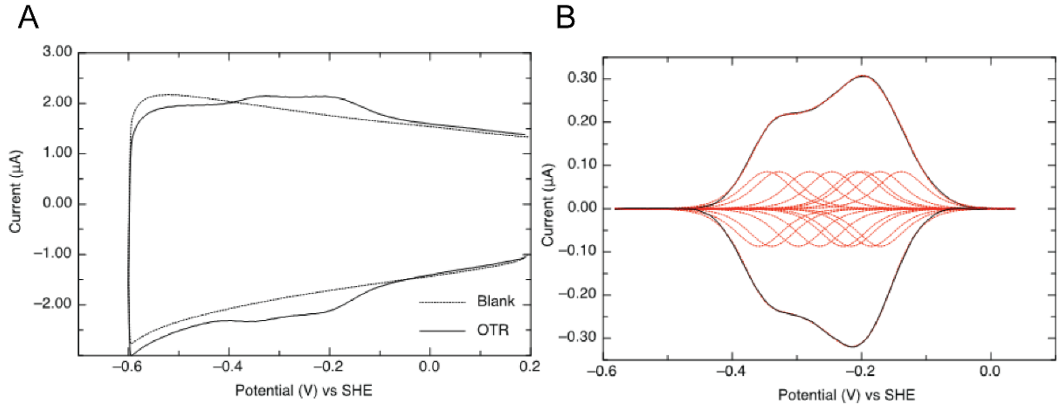

Supplementary Figure 17: Electrochemical analysis of octaheme tetrathionate reductase (OTR) from *Shewanella oneidensis*. (**A**) Non-turnover OTR voltammogram (black solid). Dotted line shows the ITO electrode baseline. (**B**) Background-subtracted voltammogram with fitting of eight reversible redox couples (red dotted). Cyclic voltammetry (CV) recorded at pH 7.4, 10°C, and a scan rate of 20 mV/s.

Supplementary Table 1: Free energy change associated with methanogenesis and iron respiration

Reaction	Electron donor	Electron acceptor	ΔG$^{0'}$ per mol substrate
$CH_3OH \rightarrow \frac{3}{4} CH_4 + \frac{1}{4} HCO_3^- + \frac{1}{4} H^+$	CH_3OH	CH_3OH	-78.75 kJ/mol
$CH_3OH + 6Fe^{3+} \rightarrow HCO_3^- + 6Fe^{2+} + 7H^+$	CH_3OH	Fe^{3+}	-665.25 kJ/mol
$CH_3COOH \rightarrow CH_4 + HCO_3^- + H^+$	CH_3COOH	CH_3COOH	-36 kJ/mol
$CH_3COOH + 8Fe^{3+} \rightarrow 2HCO_3^- + 8Fe^{2+} + 8H^+$	CH_3COOH	Fe^{3+}	-810.44 kJ/mol

ΔG$^{0'}$ values were calculated from (5).

Supplementary Table 2: Differential expression of *c*-type cytochromes in the Δ*mmcA* mutant relative to the parental strain (WWM60). Fold change >1 indicates genes that have higher expression in the Δ*mmcA* mutant compared to the parent whereas fold change <1 indicates genes that have lower expression in the Δ*mmcA* mutant compared to the parent. Genes whose expression is significantly different (q-value <0.01) are highlighted in red.

Gene id	Locus tag	Fold change	q-value
MA_RS03460	**MA0658**	**0.000451**	**4.69E-25**
MA_RS19485	**MA3739**	**1.795434**	**3.03E-08**
MA_RS15245	MA2908	1.528143	0.089088
MA_RS15315	MA2925	1.432477	0.019709
MA_RS00905	MA0167	1.742868	0.042515

Gene expression was analyzed using data from (6).

Supplementary Table 3: List of *Methanosarcina acetivorans* strains used in this study

Strains	Genotype	Source
WWM60	Δ*hpt*::P*mcrB*-*tetR*	(7)
DDN009	Δ*hpt*::P*mcrB*-*tetR*, Δ*mmcA*	(8)
DDN016	DDN009/ pDN409 [*PmcrB(tetO4)-mmcA- C- TAP tag*]	(8)
DDN037	DDN009/pDPG010 [[*PmcrB(tetO4)-uidA*]	(8)
DDN038	WWM60/pDPG010 [*PmcrB(tetO4)-uidA*]	(8)
DDN039	WWM60/pDN409 [*PmcrB(tetO4)-mmcA*-C- TAP tag]	(8)

References

1. Thauer RK, Kaster AK, Seedorf H, Buckel W, Hedderich R. 2008. Methanogenic archaea: Ecologically relevant differences in energy conservation. Nat Rev Microbiol 6, 579–591.

2. Buan NR and Metcalf WW. 2010. Methanogenesis by *Methanosarcina acetivorans* involves two structurally and functionally distinct classes of heterodisulfide reductase. Mol Microbiol 75, 843-853.

3. Lieber DJ, Catlett J, Madayiputhiya N, Nandakumar R, Lopez MM, Metcalf WW, Buan NR. 2014. A multienzyme complex channels substrates and electrons through acetyl-CoA and methane biosynthesis pathways in *Methanosarcina*. PLoS One 9, e107563.

4. Mowat CG, Rothery E, Miles CS, McIver L, Doherty MK, Drewette K, Taylor P, Walkinshaw MD, Chapman SK, Reid GA. 2004. Octaheme tetrathionate reductase is a respiratory enzyme with novel heme ligation. Nat Struct Mol Biol 11, 1023–1024.

5. Decker K, Jungermann K, Thauer, RK. 1970. Energy production in anaerobic organisms. Angew Chem Int Ed Eng 9,138-58.

6. Downing BE, Gupta D, Nayak DD. 2023. The dual role of a multi-heme cytochrome in methanogenesis: MmcA is important for energy conservation and carbon metabolism in *Methanosarcina acetivorans*. Mol Microbiol 119, 350–363.

7. Guss AM, Rother M, Zhang JK, Kulkarni G, Metcalf WW. 2008. New methods for tightly regulated gene expression and highly efficient chromosomal integration of cloned genes for *Methanosarcina* species. Archaea 2, 193-203.

8. Gupta D, Shalvarjian KE, Nayak DD. 2022. An Archaea-specific *c*-type cytochrome maturation machinery is crucial for methanogenesis in *Methanosarcina acetivorans*. ELife 11, e76970.
