# Supplementary material for: MmcA is an electron conduit that facilitates both intracellular and extracellular electron transport in Methanosarcina acetivorans
Source: Nat Commun. 2024 Apr 17;15:3300. doi: 10.1038/s41467-024-47564-2 (PMC11024163; doi:10.1038/s41467-024-47564-2)

Figure1A_
Coomassie gel

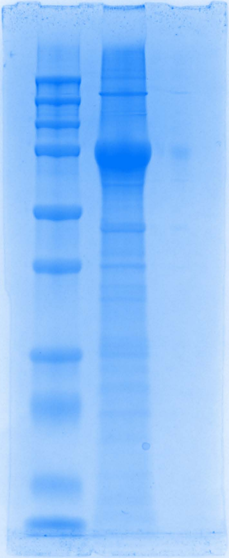

Figure1A_
Heme stain blot

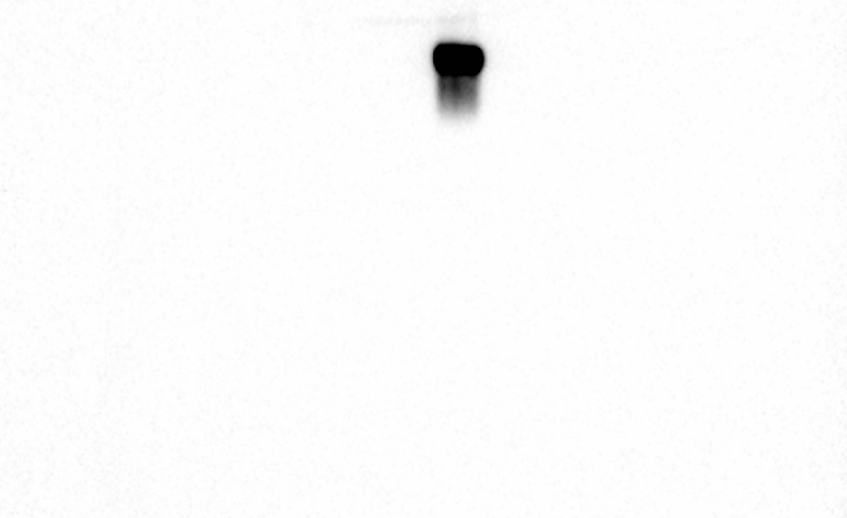

Figure1A_
Heme stain
blot_merged file

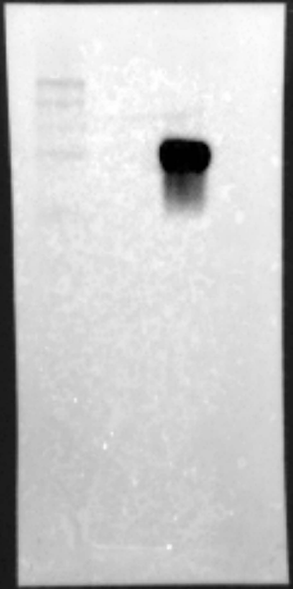

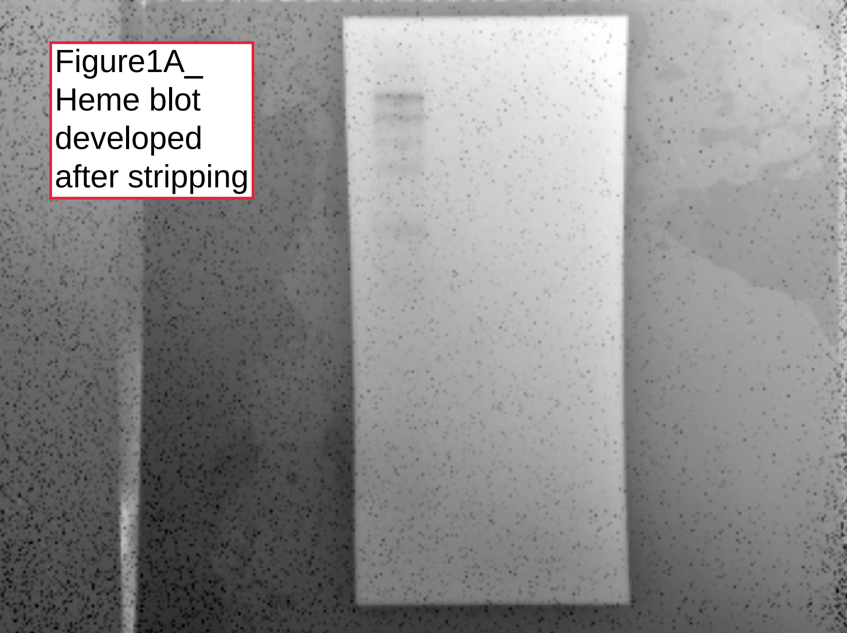

Figure1A_ Heme blot developed after stripping

Figure1A_
Western blot

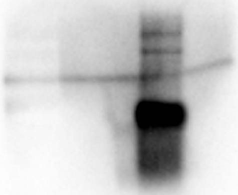

Figure1A_
Western blot_merged
file

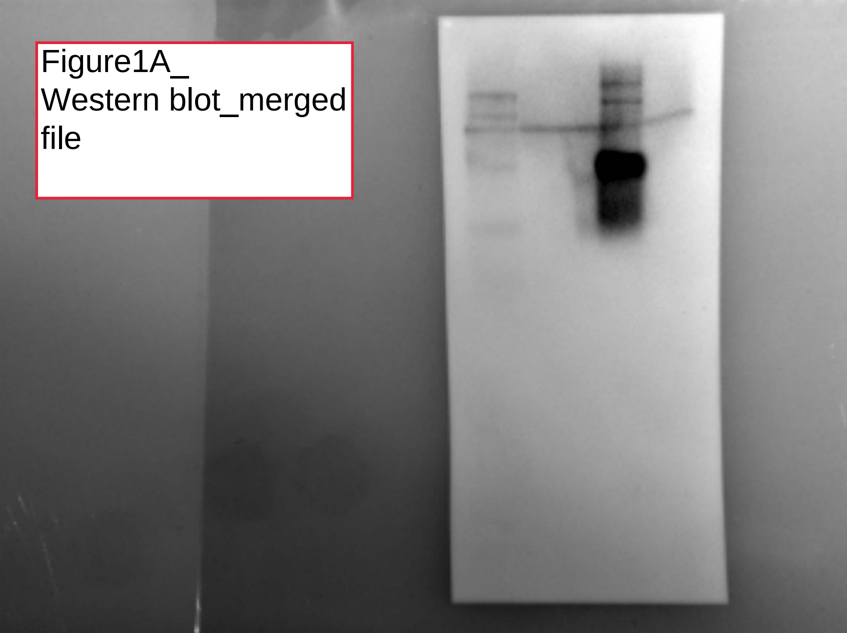

Supplement: Supplementary file 4 — Source data [file 41467_2024_47564_MOESM4_ESM.zip › Figure 1A.pdf]
