# Supplementary material for: MmcA is an electron conduit that facilitates both intracellular and extracellular electron transport in Methanosarcina acetivorans
Source: Nat Commun. 2024 Apr 17;15:3300. doi: 10.1038/s41467-024-47564-2 (PMC11024163; doi:10.1038/s41467-024-47564-2)
Supplement: Supplementary file 4 — Source data [file 41467_2024_47564_MOESM4_ESM.zip › Figure 4A.pdf]

Figure 4A_Coomassie gel

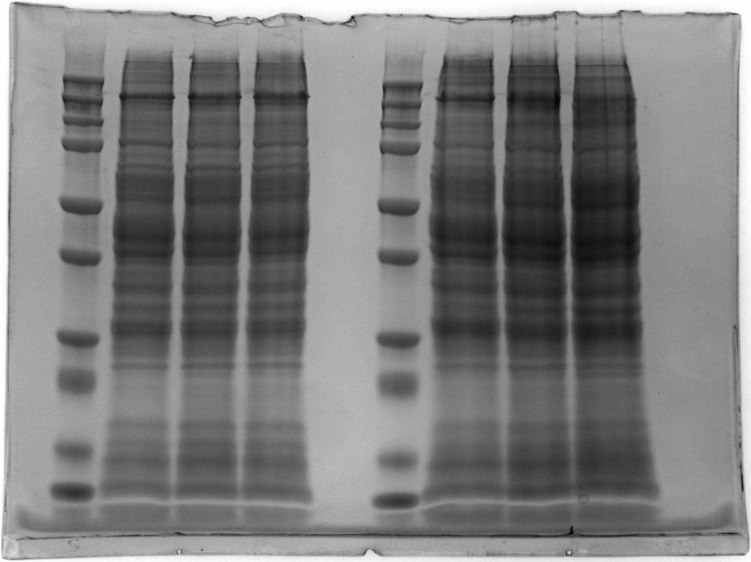

Figure 4A_Heme stain

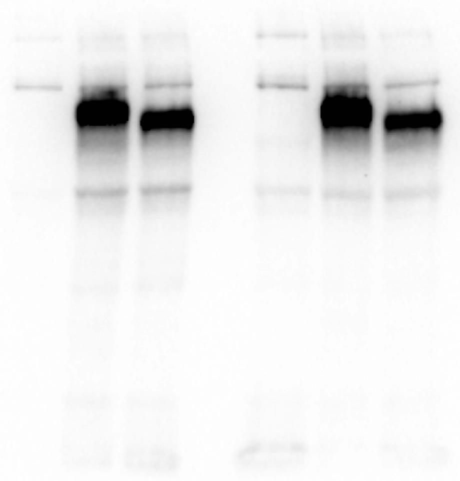

Figure 4A_Heme stain blot_merged file

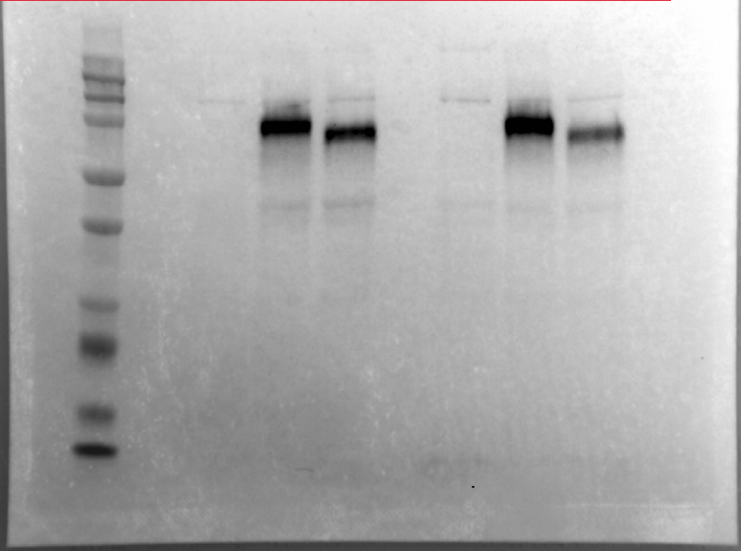

Figure 4A_heme blot developed after stripping

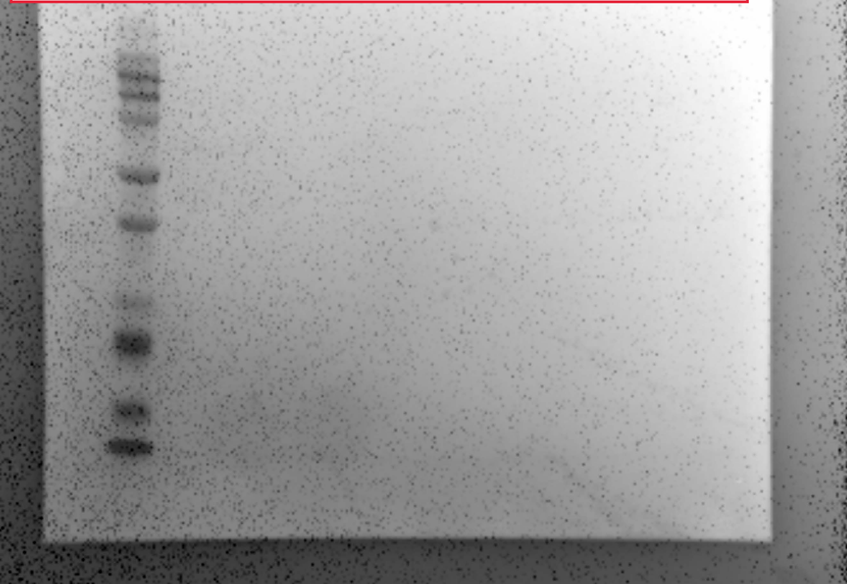

Figure 4A_anti-Flag WB

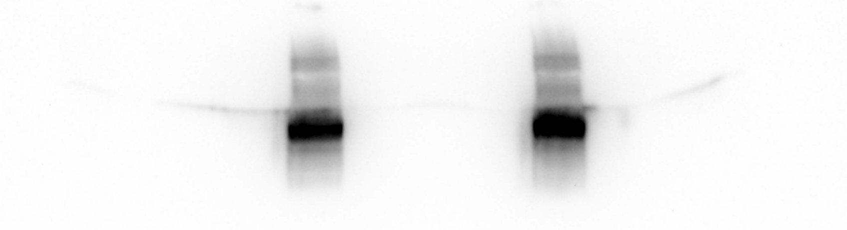

Figure 4A_anti-Flag WB_merged file

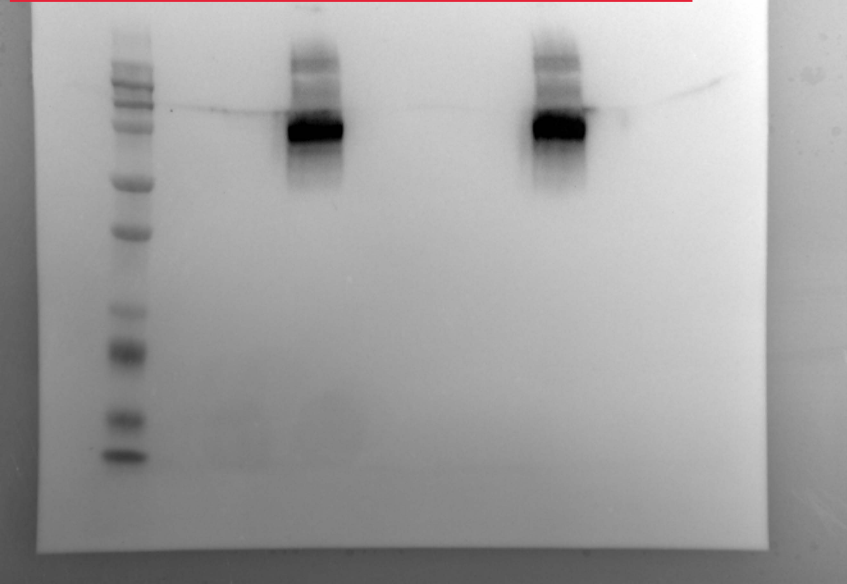

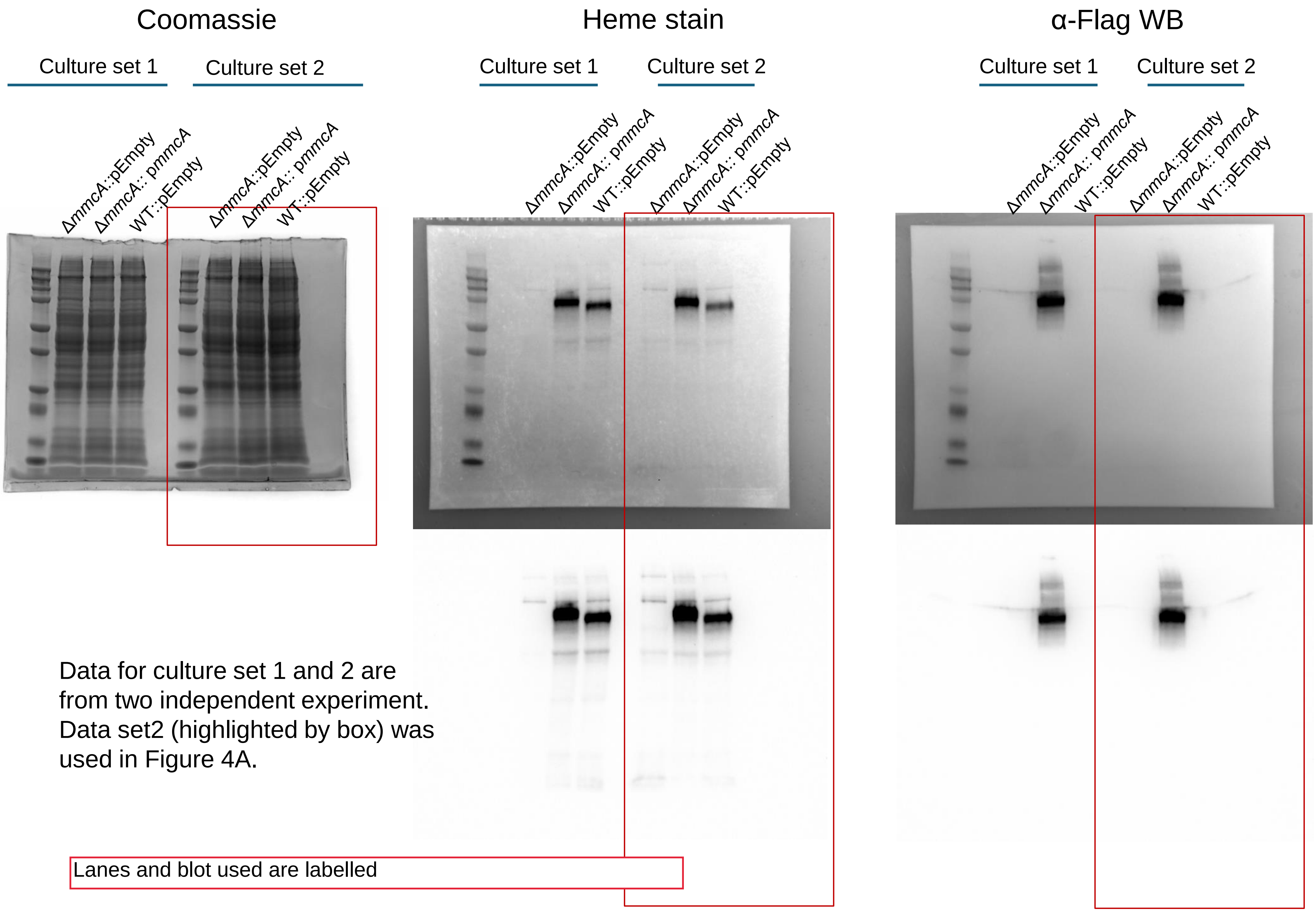

Data for culture set 1 and 2 are from two independent experiment. Data set2 (highlighted by box) was used in Figure 4A.

Lanes and blot used are labelled
