# Supplementary material for: MmcA is an electron conduit that facilitates both intracellular and extracellular electron transport in Methanosarcina acetivorans
Source: Nat Commun. 2024 Apr 17;15:3300. doi: 10.1038/s41467-024-47564-2 (PMC11024163; doi:10.1038/s41467-024-47564-2)
Supplement: Supplementary file 4 — Source data [file 41467_2024_47564_MOESM4_ESM.zip › Supplementary Figure 5A.pdf]

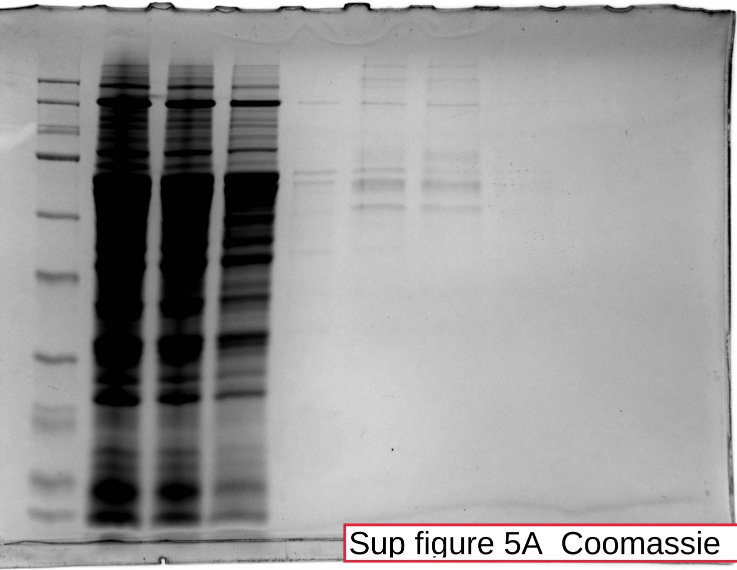

Sup figure 5A Coomassie

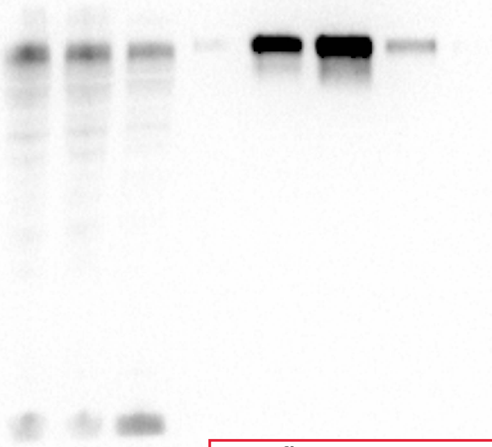

Sup figure 5A_Heme stain

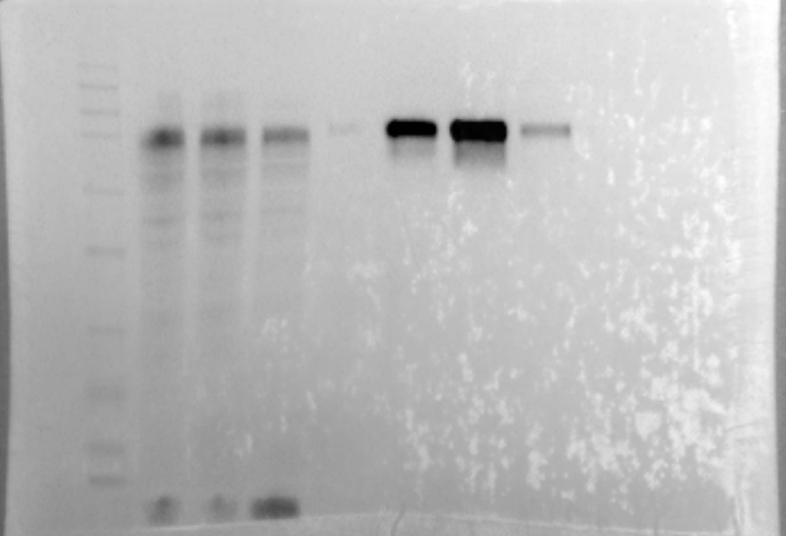

Sup figure 5A Heme stain merged

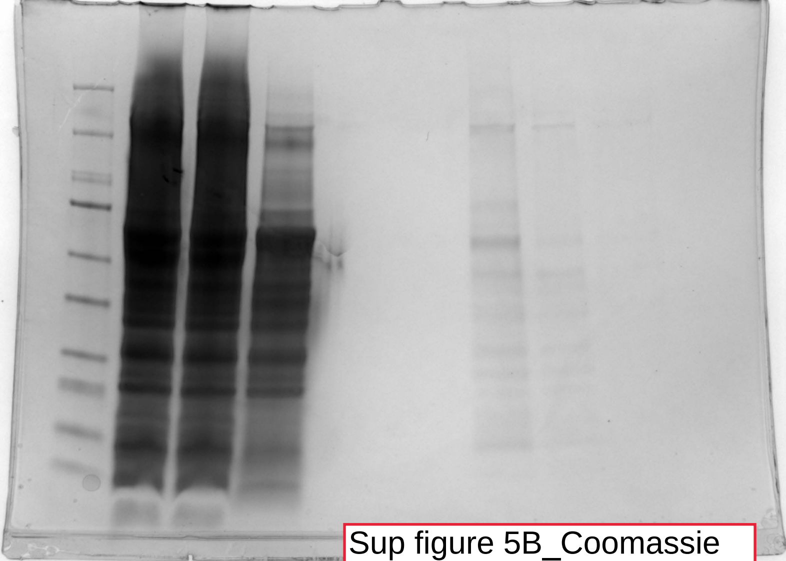

Sup figure 5B_Coomassie

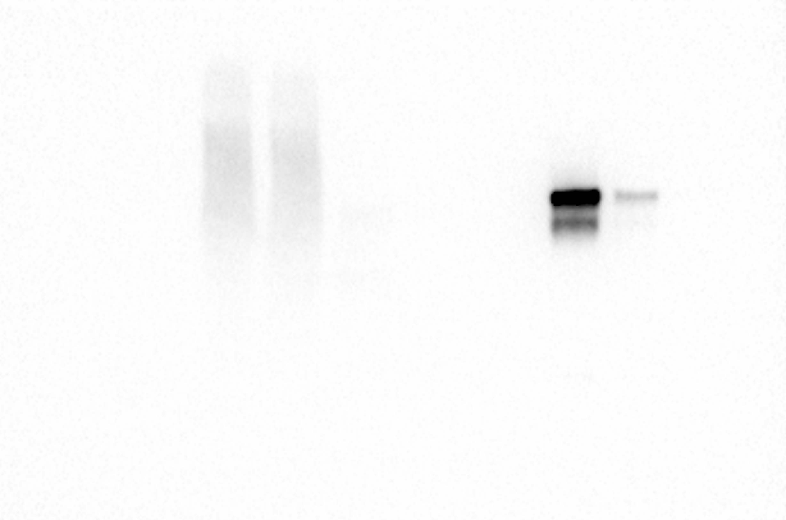

Sup figure 5B_Heme stain

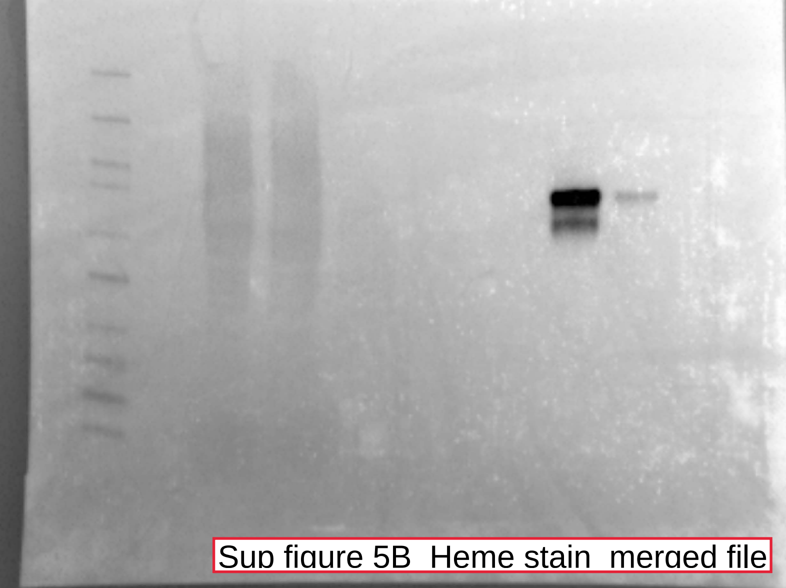

Sup figure 5B Heme stain merged file
