# Supplementary material for: MmcA is an electron conduit that facilitates both intracellular and extracellular electron transport in Methanosarcina acetivorans
Source: Nat Commun. 2024 Apr 17;15:3300. doi: 10.1038/s41467-024-47564-2 (PMC11024163; doi:10.1038/s41467-024-47564-2)

Supplementary Figure 6: Coomassie Gel

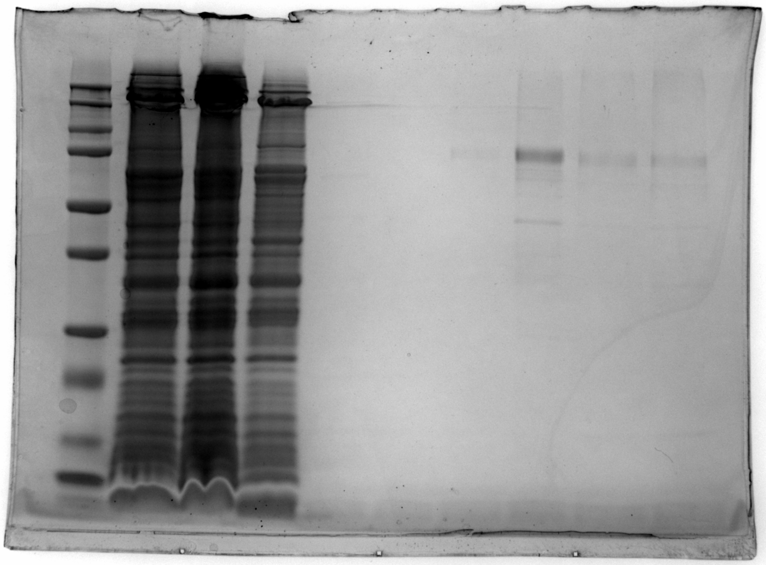

Supplementary Figure 6: Heme strain

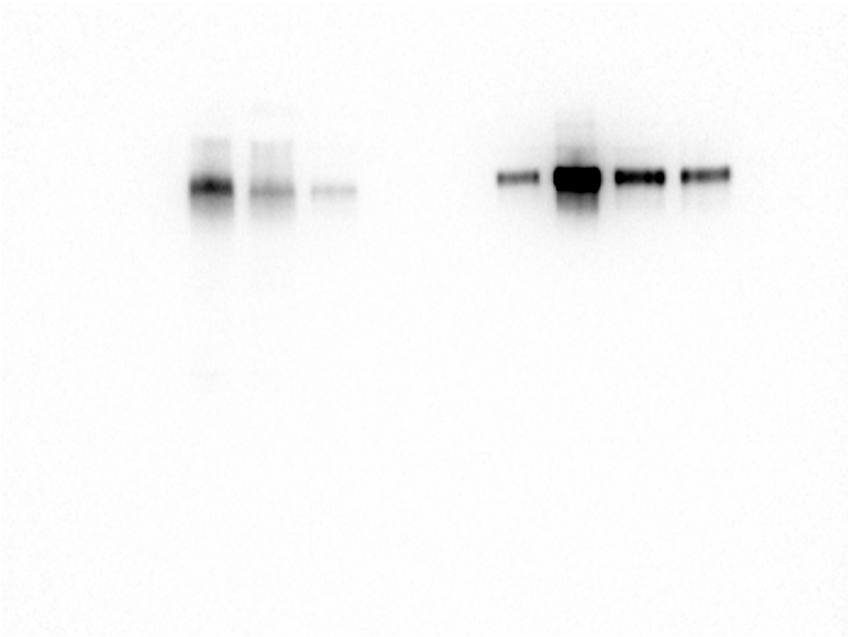

Supplementary Figure 6: Hemestrain_merged file

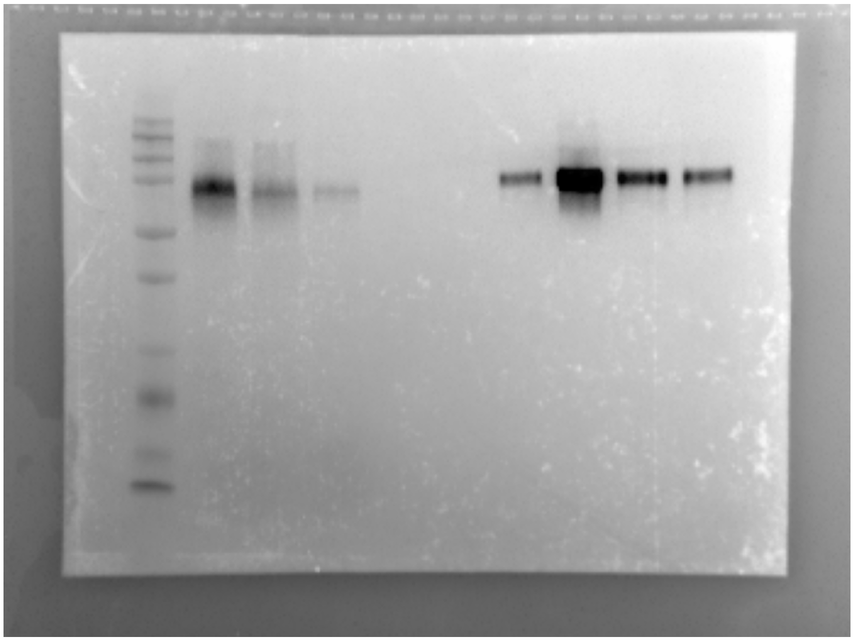

Supplement: Supplementary file 4 — Source data [file 41467_2024_47564_MOESM4_ESM.zip › Supplementary Figure 6.pdf]
